# Alabama Dental Association

**Published:** 1872-02

**Authors:** E. G. Wheeler


### ARTICLE V.

### *Alabama Dental Association.*

BY DR. E. G. WHEELER.

The third annual meeting of this Association, was held in Mobile, August 16th, 1871, the first Vice President, Wm. Deason in the chair. The roll being called the following members answerəd to their names:

Drs. Wm. Deason, J. H. Keyes, J. G. McAuley, A. H. Walker, and Robert A. Savage.

On motion the reading of the Constitution and By-Laws was dispensed with.

The minutes of the previous meeting were read and approved.

On motion the chair appointed Drs. McAuley, Keyes, and Walker, a committee on membership.

The election of members being next in order, the applications of Drs. T. H. Shaw, E. G. Wheeler, and John C. Storey were read, and being favorably reported on, they were elected members of the Association.

An adjournment was then had till 4 P. M., and upon reassembling, the subject of "Dental Education" was discussed for several hours.

Adjourned to meet Thursday morning at 10 o'clock.

The Association was called to order at 10 o'clock A. M. J. H. Keyes, second Vice President in the chair.

The minutes of the previous meeting were read and approved.

The subject of "Filling Teeth" was taken up and discussed for some time. An interesting paper was read on this subject by Dr. Wheeler and elicited much attention; a copy of which was requested for publication.

On motion of Dr. McAuley, the chair appointed Drs. Shaw, Wheeler, and Storey, a committee to draft suitable resolutions in memory of Dr. J. F. Knight of Marion, deceased.

The chair appointed Drs. Shaw, Wheeler, and Savage to procure certificates of membership.

The Association then adjourned till 4 P. M.

The Association was called to order at 4 P. M., the first Vice President, Wm. Deason, in the chair.

The committee appointed to draft resolutions in memory of Dr. J. F. Knight, presented the following:

*Whereas*, It has pleased Almighty God to remove from our midst, one of our members, Dr. J. F. Knight, of Marion, Alabama, and

*Whereas*, It becomes us to pay a worthy tribute of respect to one who has so long been identified with the profession, be it therefore

*Resolved*, That in the death of Dr. J. F. Knight, we recognize the inscrutable hand of an all-wise Providence, and that it is with sorrow we mourn his loss, yet we bow in humble submission to the decree that has deprived us of one of our most efficient and promising members. To a gifted mind were added the accomplishments of a long and systematic course of study, and the highest interest of the gentleman were sweetly blended with the graces of the christian.

Such rare combinations eminently fitted him for his professional duties, as well as to become thè devoted husband, the affectionate brother, and sympathizing friend, and we fervently trust that our loss has been his eternal gain.

*Resolved*, That a copy of these resolutions be sent to his family, and that they be spread upon the minutes of the Association.

The election of officers being next in order, the following were elected to serve for the ensuing year:

*President*—J. H. Keyes of Montgomery.

1*st Vice President*—J. C. Storey, of Eutaw.

2*d Vice President*—W. D. Dunlap, of Selma.

*Recording Secretary and Treasurer*—Robert A. Savage, of Mobile.

Delegate to Southern Dental Association— J. G. McAuley, of Selma.

Delegates to the American Dental Association—Samuel Rambo, of Montgomery; Wm. Deason, of Mobile ; W. D. Dunlap, of Selma.

*Executive Committee.*—J. G. McAuley, of Selma; T. H. Shaw, of Mobile; W. J. Reese, of Montgomery.

Mongtomery was selected as the next place of meeting. The application for membership of Dr. Geo. H. Taylor, being presented, and favorably reported on, he was elected a member of the Association. No other business being before the Association, it adjourned to meet in Montgomery on the third Wednesday in August next.

---

